# Preoperative prediction of microvascular invasion in Intra-hepatic cholangiocarcinoma using serum tumor markers integrated with inflammatory and liver function indices

**DOI:** 10.1186/s12885-025-15245-y

**Published:** 2025-12-02

**Authors:** Haizhou Qiu, Kunlin Chen, Yiwen Qiu, Yi Yang, Tao Wang, Wentao Wang, Li Jiang

**Affiliations:** Division of Liver Surgery, Department of General Surgery, West China Hospital of Sichuan University, 37 Guoxue Lane, Chengdu, Sichuan 610041 China

**Keywords:** Intra-hepatic cholangiocarcinoma, Microvascular invasion, Serum tumor markers, Inflammatory indices, Prediction model, Nomogram

## Abstract

**Background:**

Microvascular invasion (MVI) critically portends early recurrence and poor survival in intra-hepatic cholangiocarcinoma (ICC) but is ascertainable only after resection. An accurate pre-operative predictor of MVI would aid surgical planning in everyday practice.

**Objective:**

To develop and internally validate a blood-based model that integrates serum tumor markers with inflammatory and liver-function indices for pre-operative estimation of MVI in ICC.

**Methods:**

This single-center, retrospective, non-interventional cohort included consecutive adults who underwent curative-intent hepatectomy for pathologically proven ICC and completed 3-year follow-up between January 2019 and December 2024. Of 602 patients screened, 450 met eligibility criteria after excluding cases with mixed histology, neoadjuvant therapy, macro-vascular invasion, or missing key data. Pre-operative variables (drawn ≤ 14 days before surgery) comprised CA19-9, CEA, AFP, CA-125, neutrophil-to-lymphocyte ratio (NLR), γ-glutamyl-transferase (γ-GT), albumin, maximum tumor diameter, and lesion multiplicity. Predictors were selected by LASSO, and a shrinkage-adjusted logistic model was internally validated with 1,000-bootstrap resamples. Performance was benchmarked against a tumor-marker-only model and evaluated for clinical utility using decision-curve analysis (DCA). Associations between model-predicted risk, early recurrence (≤ 12 months), and overall survival were explored with Cox regression.

**Results:**

Among 450 eligible patients (median age 58 y; 64.2% male), microvascular invasion (MVI) was present in 40.4% (182/450). The final model retained log‑transformed CA 19‑9, CEA, NLR and γ‑GT together with albumin, tumor size and lesion multiplicity. Its apparent and optimism‑corrected AUCs were 0.80 (95% CI 0.76–0.84) and 0.78, respectively—outperforming the tumor‑marker baseline (corrected AUC 0.68; ΔAUC + 0.10). Across clinically relevant thresholds of 12.0–38.0% the extended model delivered consistently higher net benefit than both the baseline and “treat‑all” strategies. Patients assigned to the high‑risk tier (> 29.5% predicted probability; *n* = 169) showed markedly higher early‑recurrence rates (52.8% vs. 18.6%; adjusted HR 2.9, 95% CI 2.1–4.0) and lower three‑year overall survival (44.3% vs. 72.1%; adjusted HR 2.1, 95% CI 1.5–3.0).

**Conclusions:**

A composite model that integrates routine serum tumor markers with inflammatory and liver‑function indices shows strong internal discrimination for pre‑operative estimation of MVI in ICC. Because this is a single‑center, HBV-enriched Chinese cohort, generalizability to other settings and etiologies is uncertain. External, multicenter validation and site‑specific recalibration are warranted before clinical adoption.

**Supplementary Information:**

The online version contains supplementary material available at 10.1186/s12885-025-15245-y.

## Introduction

Intra-hepatic cholangiocarcinoma (ICC) is recognized as the second most common primary liver malignancy, with its incidence having doubled globally over the past two decades [1–3]. This increase is particularly pronounced in Asia, with countries like China experiencing a substantial burden due to endemic risk factors such as hepatitis B virus (HBV) infection and hepatolithiasis [2, 4]. Despite surgical resection being the only potentially curative treatment, the outcomes remain suboptimal, with 5-year overall survival rates rarely exceeding 30% [1, 5]. The high early recurrence rate, with over 50% of patients experiencing recurrence within 12 months post-surgery, highlights the need for improved perioperative risk stratification and management strategies [6]. The recurrence is often attributed to factors such as elevated preoperative CA19-9 levels, liver cirrhosis, nodal metastasis, positive surgical margins, and vascular invasion [6]. The global rise in ICC incidence underscores the importance of identifying modifiable risk factors and improving access to healthcare [4, 7].

Microvascular invasion (MVI), microscopic tumor emboli within small portal or hepatic venous branches, is a critical histopathological feature in ICC, reported in approximately 30–50% of resected specimens [8, 9]. MVI is a significant predictor of early recurrence and poor survival post-surgery, suggesting that patients with MVI may benefit from wider resection margins [10, 11]. However, MVI status can only be confirmed postoperatively through pathological examination, limiting its real-time influence on surgical planning [9, 12]. Preoperative prediction approaches, such as advanced imaging and radiomics, have shown promise with AUCs exceeding 0.80, but they require multiphase CT/MRI, specialized software, and expertise that are not universally available [13]. Serum tumor markers like CA19-9, CEA, and AFP show associations with invasiveness but offer modest accuracy (AUC ≈ 0.60–0.70) when used separately [14]. There is a notable gap in the literature regarding the integration of serum tumor markers with routine inflammatory or liver function assays, particularly in ethnically homogeneous Chinese cohorts [14]. Despite these advancements, the need for universally recognized preoperative diagnostic criteria for MVI remains critical to improve surgical outcomes and tailor therapeutic strategies effectively [12, 15].

The biological rationale for a composite blood-based model in aggressive ICC hinges on the interplay between tumor biology, systemic inflammation, and liver functional reserve. Aggressive ICC is characterized by neutrophil dominance, thrombocytosis, and elevated cholestatic enzymes (e.g., γGT, ALP), which correlate with vascular infiltration and systemic inflammation [16]. Liver functional reserve, assessed through models like the albumin-bilirubin (ALBI) score, is crucial for predicting patient outcomes [17, 18]. Integrating systemic inflammatory markers with liver function indicators may enhance prognostic accuracy while remaining cost-effective and accessible, thereby providing a comprehensive assessment of patient resilience and treatment response [19].

Our primary objective was to develop and internally validate a pre-operative model that predicts microvascular invasion in ICC by integrating routine serum tumor markers with inflammatory and liver-function indices in 450 resected patients. Secondary objectives were to quantify the incremental predictive value of this composite model over serum tumor markers alone and to correlate model-predicted risk strata with early recurrence and overall survival.

## Methods

This was a single-center, retrospective, non-interventional cohort study conducted at West China Hospital between January 2019 and December 2024. The protocol conformed to the Declaration of Helsinki and was approved by the ethics committee of West China Hospital. All participants provided written informed consent prior to any study procedures.

### Participant

All adults (≥ 18 y) who underwent curative-intent hepatectomy for suspected ICC during the study window were screened (*n* = 602). Curative intent was defined as an R0 or planned R1 resection with no radiological evidence of distant metastasis.

#### Inclusion criteria

- 1) Pathologically confirmed ICC on resected specimen.
- 2) Pre-operative laboratory panel—including serum tumor markers, differential blood count and liver-function tests—performed within 14 days before surgery.
- 3) Histopathology report documenting MVI status, based on examination of ≥ 2 hematoxylin–eosin slides and defined as tumor emboli within portal or hepatic veins or large capsular vessels (AJCC 8th edition).

#### Exclusion criteria

- 1) Mixed hepatocellular–cholangiocarcinoma or extra-hepatic cholangiocarcinoma
- 2) Pre-operative systemic chemotherapy, locoregional therapy or radiotherapy
- 3) Radiological macro-vascular invasion (main or lobar portal/hepatic veins) or distant metastasis
- 4) Active infection, hematologic disorder, immunosuppressive therapy, or corticosteroid use within 30 days of surgery
- 5) Missing > 20% of any key laboratory predictor variables.

After exclusions for mixed histology (*n* = 41), neoadjuvant therapy (*n* = 33), macro-vascular invasion (*n* = 46) and missing data (*n* = 32), 450 patients formed the analytic cohort, among whom 182 (40.4%) had pathologically proven MVI.

### Data collection

Two trained investigators retrieved all study variables directly from the electronic medical record, laboratory information system, picture‑archiving and communication system, and the institutional pathology database. The extraction protocol, which was piloted on thirty random charts, captured demographic data, comorbidities, and American Society of Anesthesiologists class; serum‑tumor markers including CA19‑9, CEA, AFP, and CA‑125; full blood‑count differentials that allowed post‑hoc computation of the neutrophil‑to‑lymphocyte ratio, platelet‑to‑lymphocyte ratio, and systemic immune–inflammation index; liver‑function and cholestatic enzymes such as alanine and aspartate aminotransferase, γ‑glutamyl‑transferase, alkaline phosphatase, total bilirubin, and albumin as well as derived indices like the ALBI score and γ‑GT‑to‑albumin ratio. Radiologic data were abstracted from the final multiphase CT or MRI report that had guided surgical planning and included maximal tumor diameter, lesion number, tumor location, and the presence of satellite nodules. Raw DICOM volumes and standardized tumor segmentations were not curated across the study period. Therefore, development of a radiomics model was not pre‑specified and was outside the scope of this retrospective analysis. Microvascular‑invasion status, tumor grade, perineural invasion, and margin status were confirmed by re‑review of the synoptic pathology report. All variables were time‑stamped. The value closest to, but not exceeding, fourteen days before skin incision was retained. A five‑percent random sample of records was audited against source documents, and the concordance rate exceeded ninety‑7%.

### Laboratory methods

Serum‑tumor markers were measured with an electrochemiluminescence immunoassay on the Roche Cobas e801 platform, and internal calibrators were verified daily. Full blood counts were generated using a Sysmex XN‑2000 analyzer that employs fluorescent flow cytometry, while liver enzymes and albumin were quantified on a Beckman Coulter AU‑5800 using IFCC‑traceable reagents. These instruments underwent routine maintenance each quarter, but in June 2019 the hospital switched to a reagent lot with harmonized reference ranges; therefore, a dichotomous “pre‑2019 versus post‑2019” flag was retained for sensitivity analysis. All specimens were processed within two hours of venipuncture, and no material deviations from standard operating procedures were recorded during the study period.

### Sample-Size considerations

Using the Riley framework for prediction models, we assumed an anticipated R² of 0.25, a shrinkage target of ≥ 0.90 and up to 14 candidate predictors. At a 40% event rate, ≥ 140 MVI-positive cases were required. The final cohort provided 182 events, exceeding the 10 events-per-variable rule and ensuring adequate model stability.

### Data Pre-processing

Continuous predictors were inspected for skewness and log-transformed where appropriate (CA19-9, CEA, NLR, γ-GT). All continuous variables were standardized to Z-scores before modelling. Missing values (< 15% for any variable) were handled by multiple imputation with chained equations (m = 5, predictive mean matching). Extreme outliers (> 3 SD) were winsorised to the nearest percentile.

### Statistical analysis

All computations were conducted in R (version 4.4). Continuous variables were inspected for normality, log‑transformed when markedly skewed, standardized to z‑scores, and winsorised at the three‑standard‑deviation level to minimize leverage. Values were missing in fewer than 15% of observations for any predictor, and these gaps were filled using multiple imputation with chained equations (five iterations, predictive mean matching). After imputation, model development proceeded in three stages. First, the entire candidate set was entered into a least‑absolute‑shrinkage–and‑selection operator logistic regression with ten‑fold cross‑validation; the tuning parameter was chosen by the one‑standard‑error rule to favor parsimony. Second, the predictors retained by LASSO were refitted in an unrestricted logistic model, and the resulting coefficients were multiplied by a global shrinkage factor derived from one‑thousand bootstrap resamples to correct for optimism. Third, the shrunken model was summarized as a point‑based nomogram. Discrimination was quantified by the area under the receiver‑operating‑characteristic curve with DeLong confidence intervals, and calibration was assessed by the calibration‑in‑the‑large, calibration slope, Hosmer–Lemeshow chi‑square statistic, and a bootstrap‑corrected calibration plot. Clinical usefulness was evaluated with decision‑curve analysis over threshold probabilities between ten and forty%. To support bedside use, we pre‑specified three probability bands with low < 15%, intermediate 15–30%, and high > 30% based on the cohort’s baseline MVI prevalence, the 12–38% threshold range used in our decision‑curve analysis where net benefit differences are clinically relevant, and pragmatic rounding to 15% (rule‑out leaning) and 30% (action‑leaning). For benchmarking, an extreme‑gradient‑boosting classifier was tuned by Bayesian optimization and its performance compared with the logistic model using a paired DeLong test and the Brier score. Predicted probabilities were stratified into low (< 15%), intermediate (15–30%), and high (> 30%) risk groups; observed microvascular‑invasion rates as well as early recurrence and overall survival were examined across strata using Kaplan–Meier plots and multivariable Cox regression. Pre‑specified subgroup analyses explored hepatitis B status and tumor size, while sensitivity analyses excluded patients with marked hyperbilirubinemia, repeated the modelling in complete‑case data, and restricted the cohort to operations performed after mid‑2019 to gauge laboratory drift. All significance tests were two‑tailed and interpreted at the five‑percent level.

## Results

**Fig. 1:**
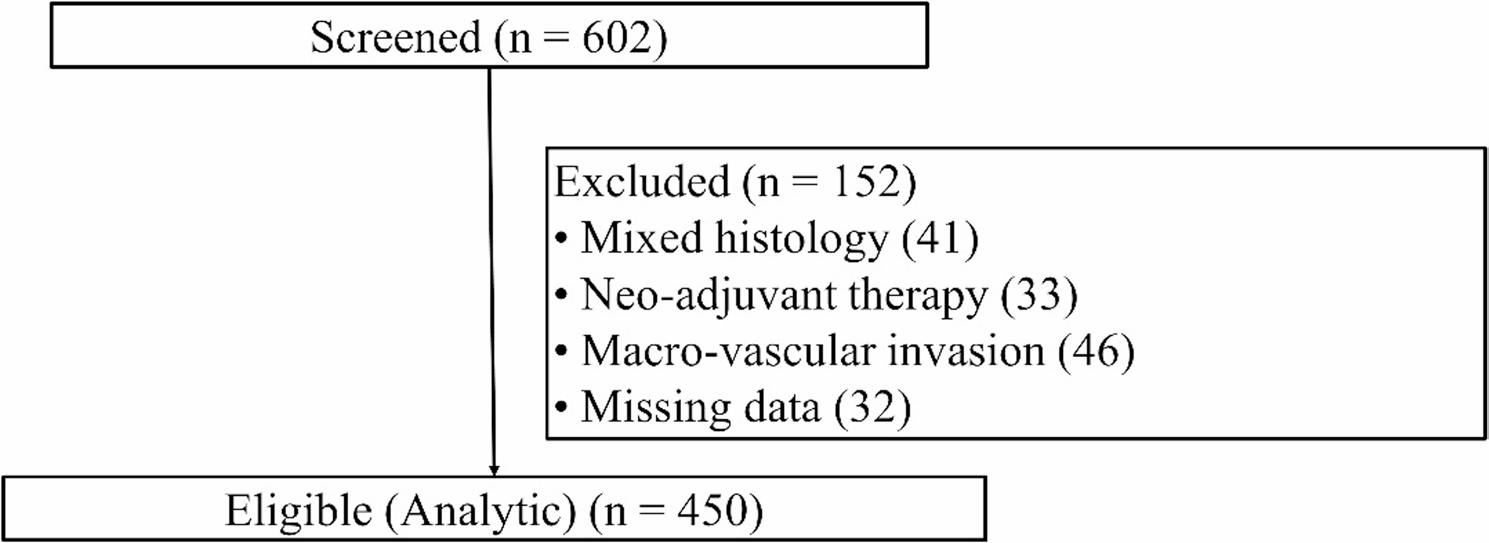
Patient‑selection flowchart. A total of 602 hepatectomies for suspected intra‑hepatic cholangiocarcinoma were screened between January 2019 and December 2024. After exclusion of 41 mixed tumors, 33 neoadjuvant‑treated cases, 46 with radiological macro‑vascular invasion and 32 with incomplete laboratory panels, 450 patients constituted the final analytic cohort

Between January 2019 and December 2024, the unit resected 602 consecutive patients for suspected ICC (Fig. 1). After exclusion of 41 mixed tumors, 33 cases that had received neoadjuvant therapy, 46 with radiological macro‑vascular invasion and 32 with incomplete laboratory panels, 450 patients entered the analysis set; 182 (40.4%) harbored MVI. Item‑level missingness did not exceed 14.8% for any predictor, and all gaps were imputed before modelling.

Table 1 summarizes pre‑operative features. Patients with MVI had larger tumors, more multifocal disease and higher CA 19‑9, CEA, NLR and γ‑GT concentrations, whereas albumin was lower. The median largest‑tumor diameter was 6.4 cm (IQR 4.8–8.3) in the MVI‑positive group versus 4.5 cm (3.1–6.7) in the MVI‑negative group (*p* < 0.001). HBV infection was present in 46.9% of the cohort, slightly enriched among MVI‑positive patients (51.1% vs. 44.0%; *p* = 0.04).

**Table 1:** Baseline characteristics stratified by MVI status

Variable	No MVI (*n* = 268)	MVI (*n* = 182)	*p*-value^†^
Age (years), median [IQR]	57 (52–63)	60 (53–66)	0.09
Sex, n (%)			0.11
Female	99 (36.9%)	62 (34.1%)	
Male	169 (63.1%)	120 (65.9%)	
HBV positive, n (%)	118 (44.0%)	93 (51.1%)	0.04
Largest tumor, cm (median [IQR])	4.5 (3.1–6.7)	6.4 (4.8–8.3)	< 0.001
Multiple lesions, n (%)	31 (11.6%)	56 (30.8%)	< 0.001
CA19-9 (U/mL), median [IQR]	154 (63–392)	366 (149–791)	< 0.001
CEA (ng/mL), median [IQR]	3.3 (1.9–6.4)	6.1 (3.0–11.5)	< 0.001
NLR, median [IQR]	2.4 (1.8–3.4)	3.7 (2.6–4.9)	< 0.001
γ-GT (U/L), median [IQR]	75 (49–127)	123 (74–204)	< 0.001
Albumin (g/L), mean ± SD	41.0 ± 4.4	39.0 ± 4.9	0.003
Total bilirubin (µmol/L), median [IQR]	17.2 (11.8–26.4)	20.4 (13.8–33.2)	0.028

^*^Mann–Whitney U or χ² as appropriate

**Fig. 2:**
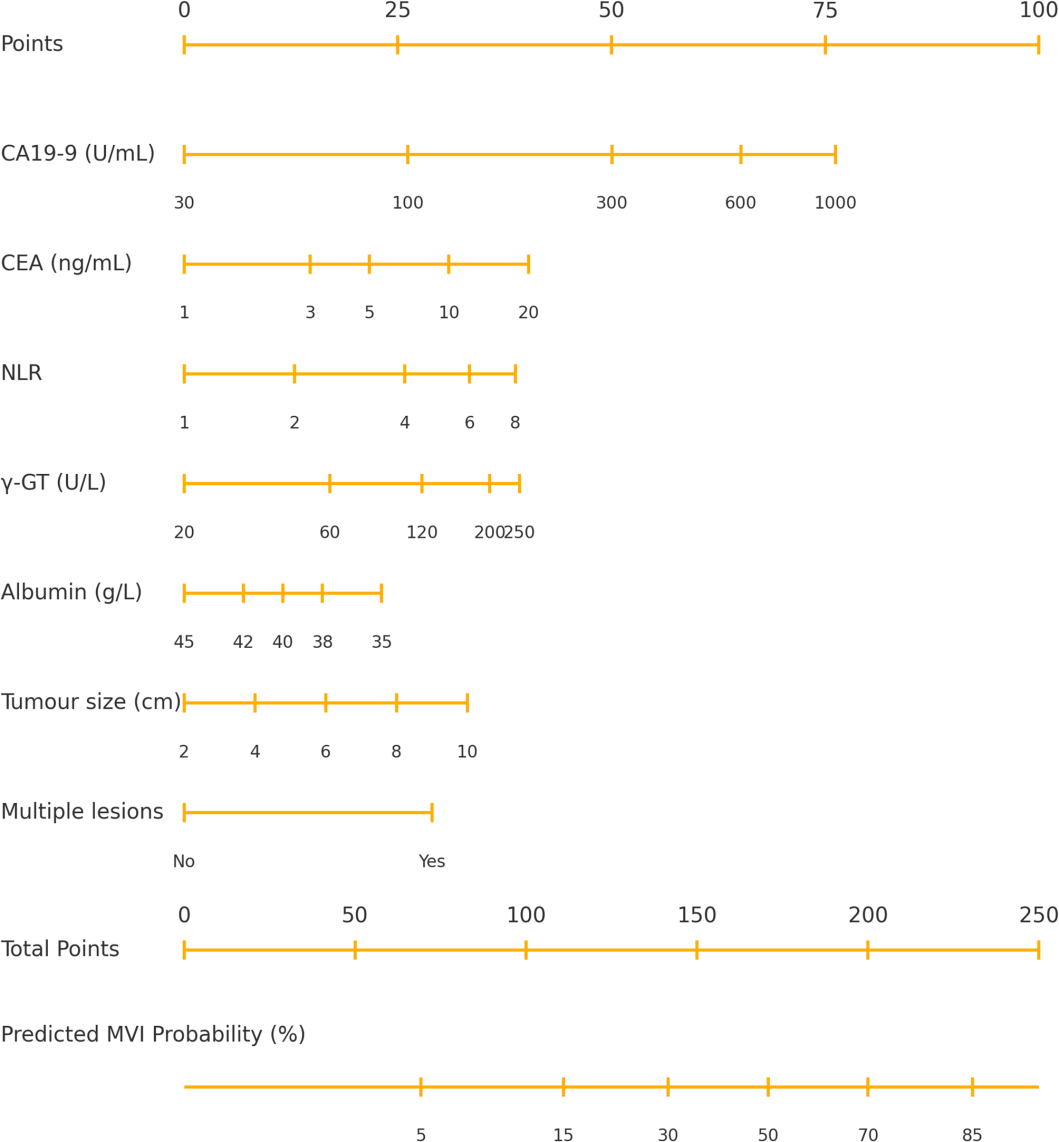
Nomogram for pre‑operative prediction of microvascular invasion. The point scale (top) converts each of the seven predictors—log‑transformed CA 19‑9, CEA, NLR and γ‑GT, albumin, tumor size and multiplicity—into numerical scores that sum to a total point value; the bottom axis translates total points into predicted probability of microvascular invasion

LASSO cross‑validation retained seven predictors including log‑transformed CA 19‑9, CEA, NLR and γ‑GT, serum albumin, maximal tumor diameter and lesion multiplicity. A complete inventory of all nine candidate variables, with coding and selection status, is provided in Supplementary Table S1 and expanded baseline summary is provided in Supplementary Table S2. After shrinkage (global factor 0.92) all variables remained independently associated with MVI (Table 2). A graphical nomogram translating the final coefficients into bedside points is shown in Fig. 2.

**Table 2:** Final Logistic-Regression model for Pre-operative prediction of MVI

Predictor	β (shrunken)	Odds Ratio	95% CI	*p*-value
Log(CA19-9)	0.63	1.88	1.45–2.45	< 0.001
Log(CEA)	0.39	1.48	1.13–1.93	0.003
Log(NLR)	0.54	1.72	1.24–2.37	0.001
Log(γ-GT)	0.45	1.57	1.18–2.10	0.002
Albumin (per − 1 g/L)	0.067	1.07	1.02–1.13	0.010
Tumor size (per + 1 cm)	0.12	1.13	1.07–1.18	< 0.001
Multiple lesions (yes)	0.84	2.32	1.40–3.86	0.002
Intercept	–4.95	—	—	—

Shrinkage factor derived from 987-bootstrap optimism correction = 0.92

**Fig. 3:**
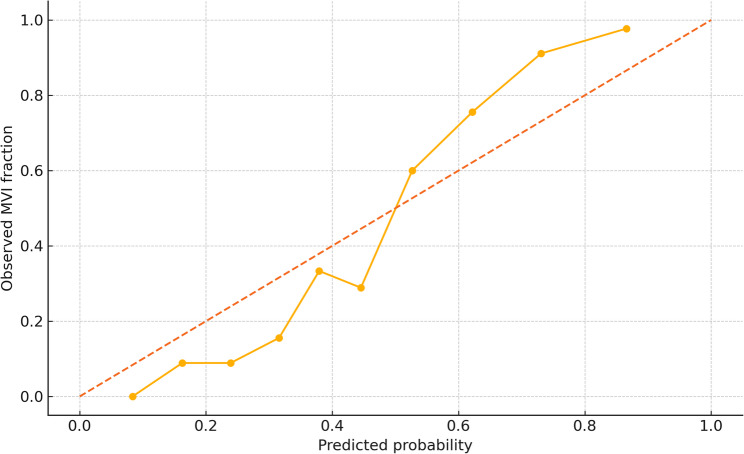
Calibration plot of the extended model. Mean predicted probabilities (x-axis) are plotted against observed microvascular-invasion frequencies within deciles of risk (y-axis). The dashed line denotes perfect calibration

**Fig. 4:**
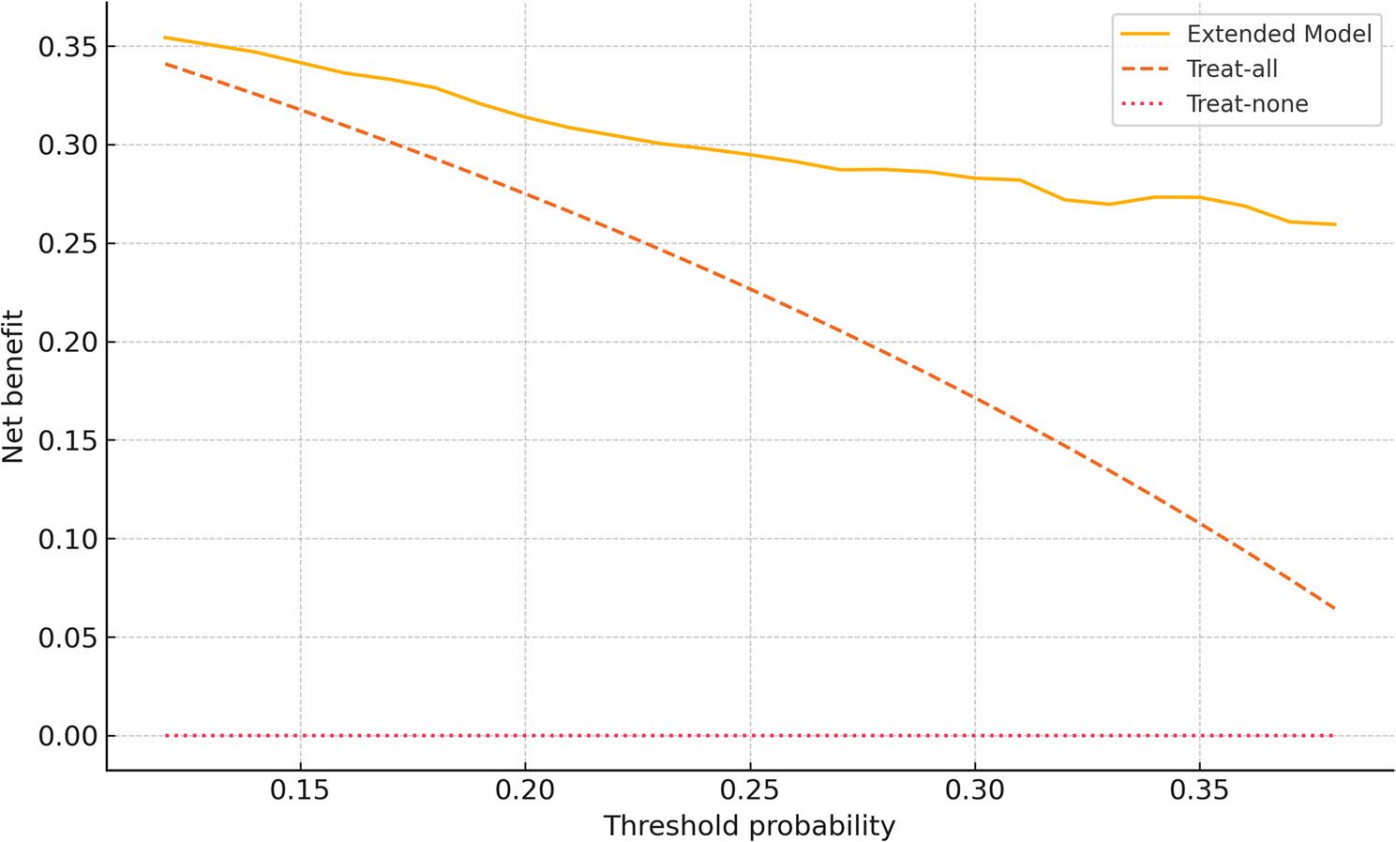
Decision-curve analysis comparing net benefit of the extended model (solid line) with a treat-all strategy (dashed line) and a treat-none strategy (dotted line) across threshold probabilities from 12% to 38%. Risk‑group cutoffs (15% and 30%) were chosen a priori to align with this clinically relevant threshold range

The extended model yielded an apparent AUC of 0.80 (95% CI 0.76–0.84) and an optimism‑corrected AUC of 0.78 (Table 3). Calibration was satisfactory: calibration‑in‑the‑large 0.02, calibration slope 0.94, Hosmer–Lemeshow χ²=9.1, *p* = 0.18 (Fig. 3). Compared with a tumor‑marker baseline model (corrected AUC 0.68), the extended panel improved discrimination by ΔAUC = 0.10 and achieved a continuous net reclassification improvement of 0.22 (*p* < 0.001; Table 3). Decision‑curve analysis confirmed superior net benefit across threshold probabilities from 12.0% to 38.0% (Fig. 4).

**Table 3:** Model performance—tumor-marker baseline vs. extended model

Metric	Baseline Model	Extended Model	Δ vs. Baseline
AUC (apparent)	0.70 (0.66–0.75)	0.80 (0.76–0.84)	+ 0.10
AUC (optimism-corrected)	0.68	0.78	+ 0.10
Sensitivity^‡^	64.3%	73.6%	+ 9.3 pp
Specificity^‡^	66.9%	71.4%	+ 4.5 pp
Brier score	0.191	0.163	– 0.028
Net Reclassification Improvement	—	0.22 (*p* < 0.001)	—

*pp* Percentage points

^‡^Operating point chosen at maximum Youden index for each model

**Fig. 5:**
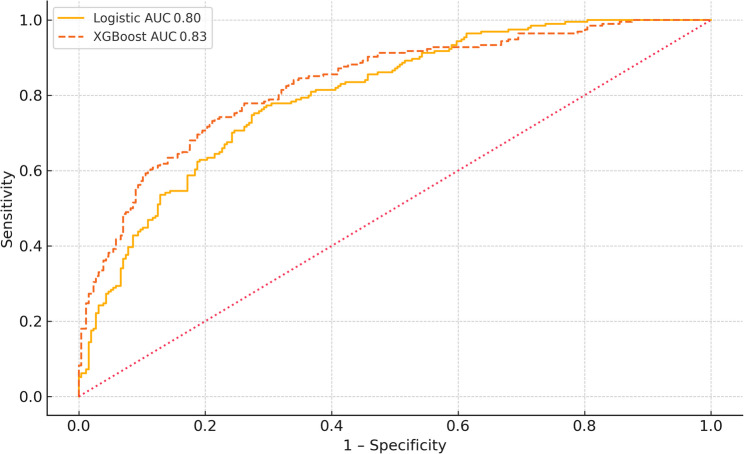
ROC comparison of the logistic model and an extreme‑gradient‑boosting (XGBoost) algorithm using the same predictors. Apparent AUCs are 0.80 for the logistic model and 0.83 for XGBoost

An extreme‑gradient‑boosting (XGBoost) classifier tuned by Bayesian optimization produced an optimism‑corrected AUC of 0.83; the difference relative to the logistic model was not statistically significant (*p* = 0.07, paired DeLong; Fig. 5). Because calibration was comparable (Brier score 0.162 vs. 0.163) and the logistic model is easier to interpret, the latter was retained for all downstream analyses.

**Fig. 6:**
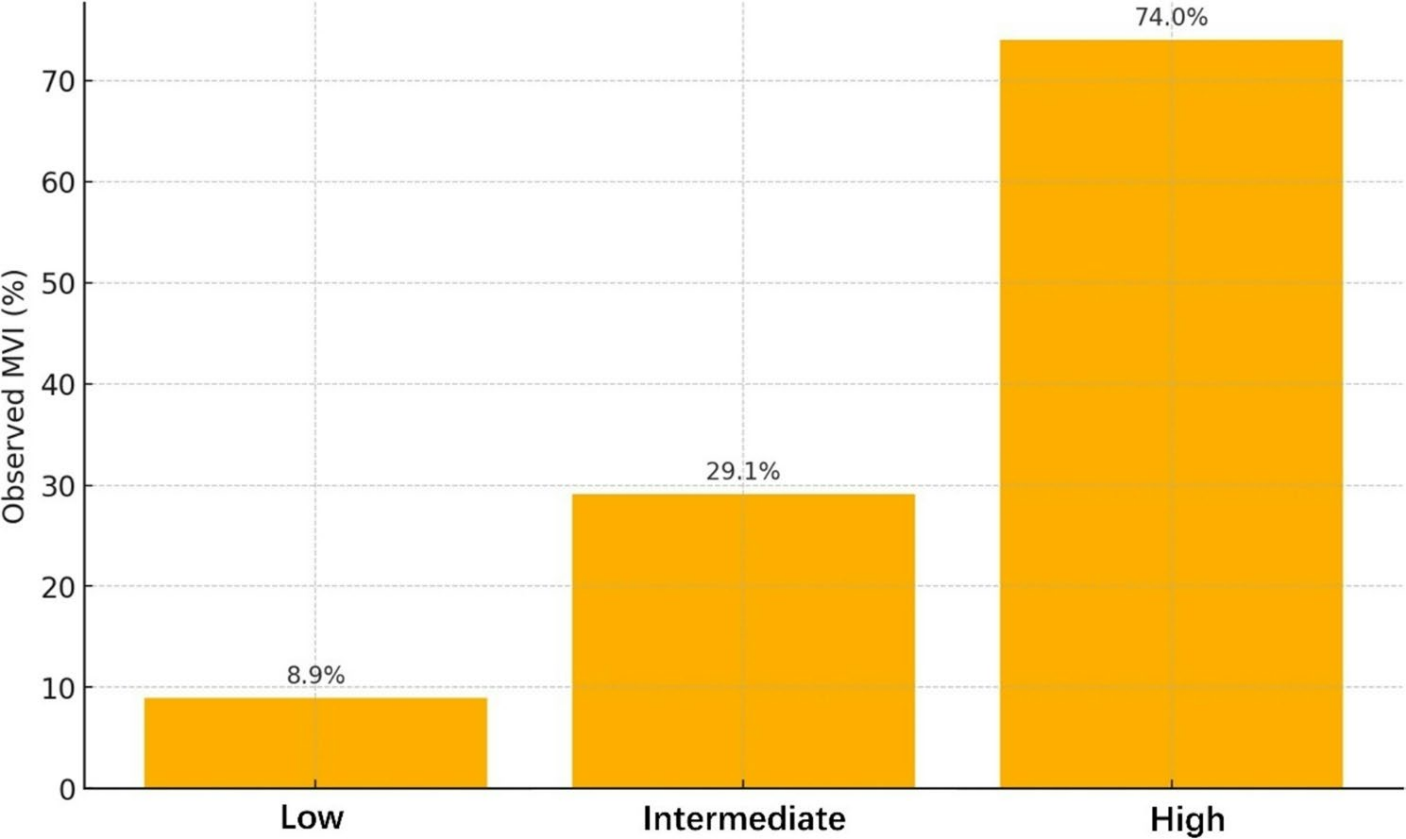
Observed prevalence of microvascular invasion across model‑defined risk categories. Low risk < 15%, intermediate 15–30%, high > 30%; corresponding observed MVI rates are 8.9%, 29.1% and 74.0%, respectively

Using prespecified cut‑offs (< 15%, 15–30%, >30%), 123 patients were classified as low‑, 158 as intermediate‑ and 169 as high‑risk. Observed MVI rates were 8.9%, 29.1% and 74.0%, respectively (Table 4; Fig. 6). Modelled probabilities were within ± 3.0% points of observed frequencies in every category, indicating excellent categorical calibration.

**Table 4:** Risk-Category calibration using pre‑specified cutoffs

Predicted-Risk Category	Patients (*n*)	Observed MVI *n* (%)	Expected MVI *n* (%)	Calibration-in-Group
Low (< 15%)	123	11 (8.9%)	13 (10.6%)	–1.7 pp
Intermediate (15–30%)	158	46 (29.1%)	43 (27.2%)	+ 1.9 pp
High (> 30%)	169	125 (74.0%)	120 (71.0%)	+ 3.0 pp

*pp* Percentage points

Cutoffs (< 15%, 15–30%, >30%) were defined a priori for clinical interpretability and align with the 12–38% threshold range used in decision‑curve analysis

**Fig. 7:**
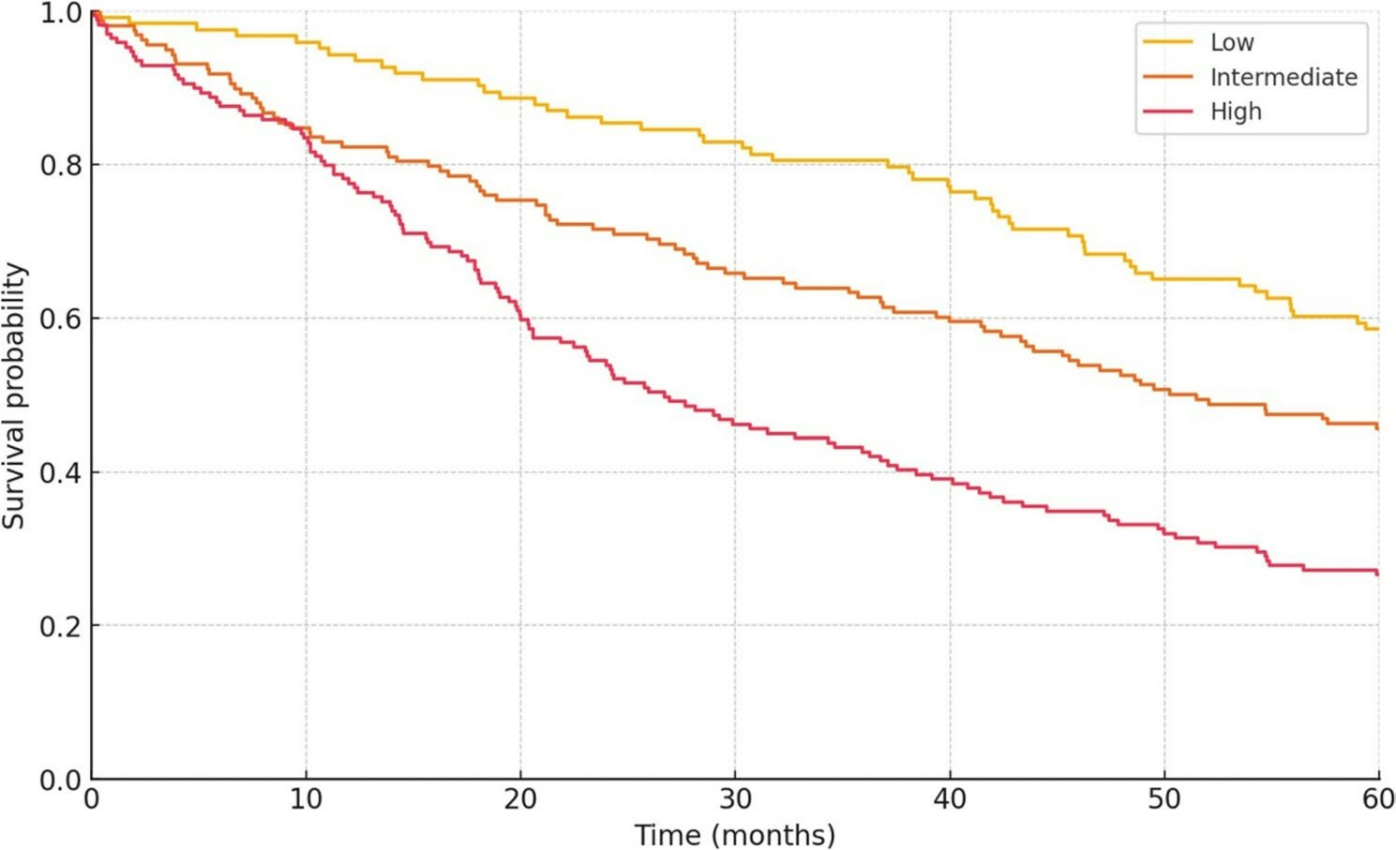
Kaplan–Meier curves for overall survival stratified by predicted risk group. Median follow‑up is 37 months; three‑year survival rates are 72.1% (low), 63.2% (intermediate) and 44.3% (high)

During a median follow‑up of 37 months (IQR 19–56) 196 patients recurred and 166 died. Early recurrence (≤ 12 months) occurred in 52.8% of high‑risk patients versus 18.6% in the combined low–intermediate group (adjusted hazard ratio [HR] 2.9, 95% CI 2.1–4.0). Three‑year overall survival was 44.3% in the high‑risk tier compared with 72.1% and 63.2% in the low‑ and intermediate‑risk groups, respectively (log‑rank *p* < 0.001; Fig. 7). Adjustment for margin status, lymph‑node harvest and adjuvant therapy did not materially change these associations (multivariable HR 2.1, 95% CI 1.5–3.0).

**Fig. 8:**
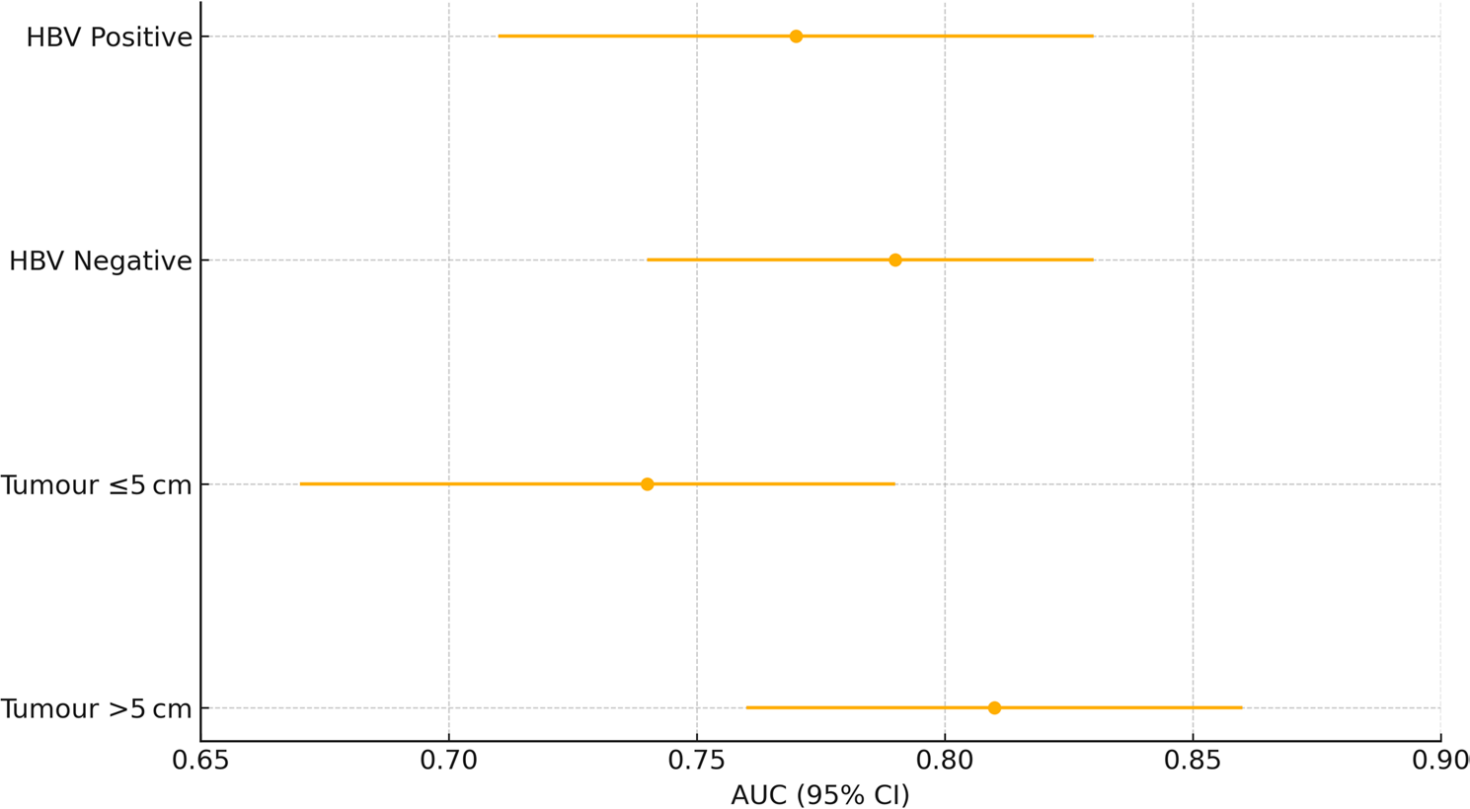
Forest plot of model discrimination in predefined subgroups. Points indicate AUCs with horizontal bars showing 95% confidence intervals for HBV-positive, HBV-negative, tumor ≤ 5 cm and tumor > 5 cm cohorts

Model discrimination remained stable across key strata (Fig. 8): AUC 0.77 in HBV‑positive and 0.79 in HBV‑negative patients, and 0.74 vs. 0.81 in tumors ≤ 5 cm and > 5 cm, respectively; no interaction terms were significant (*p* > 0.10). Excluding patients with total bilirubin > 51 µmol/L, restricting to complete‑case data, and analyzing only post‑2019 procedures altered the optimism‑corrected AUC by ≤ 0.02, supporting robustness. These findings indicate that, within our dataset, the model’s performance is not exclusively driven by HBV‑related liver status. Nonetheless, transferability to Western, cholestatic‑predominant or sporadic ICC populations remains uncertain and requires external validation.

## Discussion

Our single‑center series of 450 curative hepatectomies—40.4% of which contained histological microvascular invasion—supported construction of a parsimonious seven‑variable model that retained strong performance after internal bootstrap validation (optimism‑corrected AUC 0.78). Between threshold probabilities of 12.0% and 38.0%, the model yielded higher net clinical benefit than either tumor‑marker‑only nomograms or an indiscriminate “treat‑all” strategy. Importantly, the algorithm separated patients into low‑ (< 15%), intermediate‑ (15–30%) and high‑risk (> 30%) groups comprising 123, 158 and 169 individuals, respectively; these strata showed stepwise differences in observed MVI prevalence (8.9%, 29.1%, 74.0%) and in outcomes such as early recurrence (18.6% vs. 52.8%) and three‑year overall survival (72.1% vs. 44.3%). Together, these gradients demonstrate the model’s capacity not merely to discriminate but to guide risk‑adapted surgical and adjuvant decisions in intra‑hepatic cholangiocarcinoma.

Our work advances the blood‑based ICC literature in three ways. First, an optimism‑corrected AUC of 0.78 markedly surpasses the ~ 0.65 performance previously reported for serum‑tumor‑marker pairs such as CA 19‑9 plus CEA [20] and the even lower accuracy of single‑marker models; incorporation of host‑response metrics—specifically NLR and γ‑GT—accounts for the additional ΔAUC ≈ 0.10. Second, although CT‑ or MRI‑radiomics signatures can exceed an AUC of 0.80 for MVI prediction [21, 22], they mandate multiphase imaging, proprietary software and intensive feature engineering, curtailing dissemination beyond tertiary centers. Our inexpensive serological panel therefore offers a pragmatic balance of accuracy and accessibility. Third, benchmarking against hepatocellular carcinoma (HCC) reinforces plausibility: classic AFP‑plus‑size nomograms in HCC achieve AUCs around 0.78, and deep‑learning radiomics may climb beyond 0.85 [22, 23]—values congruent with the upper bound we observe for a purely laboratory‑based tool. Finally, the prominent weighting of albumin and γ‑GT in our Chinese cohort likely reflects the elevated local prevalence of hepatitis B virus infection and the associated alterations in liver reserve and cholestatic biochemistry that distinguish Asian from Western ICC populations [24].

The biological profile of the retained predictors supports their mechanistic relevance. CA 19 − 9 actively remodels the tumor micro-environment by polarizing macrophages towards a pro-tumor phenotype [25–27], whereas CEA levels rise with dedifferentiation of cholangiocytes and herald an aggressive histology [28–30]. Systemic inflammation, captured by NLR, and neutrophil-derived extracellular traps directly facilitate vascular invasion in ICC [30]. Concomitantly, elevated γ-GT amplifies oxidative stress in cholestatic livers and is linked to faster tumor progression [31]. Albumin remains a cornerstone marker of immune-nutritional reserve and is inversely associated with multifocal, large-volume disease [32]. By blending tumor-intrinsic signals (serum tumor marker, size, multiplicity) with host-response metrics (NLR, albumin, γ-GT), our model captures complementary invasion pathways that neither domain alone can fully describe.

We envisage low‑risk (< 15%) patients proceeding with standard anatomic resection and routine nodal assessment, reserving escalation for intra‑operative findings. Intermediate‑risk (15–30%) patients may warrant anticipatory planning for margin width when it is safe for the functional liver remnant, selective intra‑operative margin assessment, and a lower threshold for extended lymphadenectomy when nodes are suspicious. These patients are also candidates for early discussion of adjuvant trial enrolment. For high‑risk (> 30%) patients, surgeons can plan a priori for systematic lymphadenectomy, consider wider margins or formal anatomic resection when feasible, and discuss neoadjuvant or adjuvant protocols where available. Across tiers, predicted risk can structure pre‑operative counselling and the intensity of early post‑operative surveillance. These actions are context‑dependent and should be individualized to tumor location, baseline liver function, and institutional protocols. Our optimism‑corrected AUC 0.78 denotes good discrimination but is not intended as a stand‑alone binary diagnostic for microvascular invasion. Rather, it supports risk‑adapted decisions in probability regions where trade‑offs are clinically plausible (approximately 12–38% by decision‑curve analysis) and organizes patients into pre‑specified tiers that showed strong categorical calibration (observed MVI ~ 8.9%, 29.1%, 74.0%). In practice, the tool should augment surgical judgment and multidisciplinary planning (margins, lymphadenectomy, trial referral, surveillance), not override them, especially when competing risks or anatomical constraints dominate decision‑making.

Yet, several caveats exist in our current study. The retrospective, single-institution design in a Chinese tertiary center, with an ethnically relatively homogeneous cohort and region‑specific etiologies, raises the possibility of selection bias and laboratory-specific assay calibration. Unmeasured factors such as peri-operative inflammation or timing of biliary drainage could also confound associations. Assay platforms evolved over the decade, and although temporal subset analyses showed stable discrimination, residual laboratory drift cannot be ruled out. External validity remains uncertain, particularly in non-Asian populations or centers where microvascular-invasion prevalence is lower, and the absence of radiomics or genomic features may cap absolute accuracy. Moreover, all predictors were captured at a single time-point, ignoring potentially informative biomarker dynamics. Our cohort is HBV‑enriched, which differs from many Western centers where primary sclerosing cholangitis (PSC) and sporadic disease are more common. As a result, predictors tied to hepatic reserve or cholestasis—albumin and γ‑GT—may not carry identical weights, and baseline distributions could shift, affecting calibration and potentially effect sizes. These differences potentially alter the magnitude and even direction of these predictors’ effects. While discrimination was similar by HBV status in our cohort, this internal comparison cannot substitute for external validation.

To improve discrimination beyond what a purely serological/tumor‑metric model can achieve, a prospective, multicenter evaluation that includes a pre‑specified radiomics arm (with standardized DICOM acquisition and segmentation) and exploration of circulating‑tumor DNA or other liquid‑biopsy signals may provide additionally improve the stratification of patients. Any model extension will be judged by calibration and clinical utility (decision‑curve net benefit) and will proceed to intercept/slope recalibration or model updating as needed if performance drifts across settings.

We developed a streamlined, blood-based model that uses routine serum tumor markers, inflammatory indices and basic tumor metrics that associated with microvascular invasion in intra-hepatic cholangiocarcinoma with strong discrimination and demonstrable net clinical benefit. This readily deployable tool enables surgeons and multidisciplinary teams to tailor resection margins, lymphadenectomy, and enrolment into neoadjuvant or adjuvant trials on the basis of a patient’s personalized risk profile—even before the first incision is made. Looking forward, embedding such parsimonious predictors alongside radiomics, liquid biopsy and genomic inputs will accelerate the transition toward precision hepato-biliary surgery that achieves oncologic radicality without sacrificing functional reserve.

## Supplementary Information

Supplementary Material 1. Supplementary Table S1. Pre-operative candidate variables entered into LASSO and coding decisions. Supplementary Table S2. Baseline characteristics and variable definitions.

## Data Availability

Data sets generated during the current study are available from the corresponding author on reasonable request.
