# Supplementary material for: Preoperative prediction of microvascular invasion in Intra-hepatic cholangiocarcinoma using serum tumor markers integrated with inflammatory and liver function indices
Source: BMC Cancer. 2025 Dec 2;26:27. doi: 10.1186/s12885-025-15245-y (PMC12777458; doi:10.1186/s12885-025-15245-y)
Supplement: Supplementary file 1 — Supplementary Material 1. Supplementary Table S1. Pre-operative candidate variables entered into LASSO and coding decisions. Supplementary Table S2. Baseline characteristics and variable definitions. [file 12885_2025_15245_MOESM1_ESM.docx]

**Supplementary Table S1. Pre‑operative candidate variables entered into LASSO and coding decisions**

| **Domain** | **Variable (exact label)** | **Unit** | **Preprocessing for modeling** | **Entered as LASSO candidate** | **Selected in final model** |
| --- | --- | --- | --- | --- | --- |
| Tumor markers | CA 19‑9 | U/mL | log(x), then z‑score | Yes | **Yes** |
| Tumor markers | CEA | ng/mL | log(x), then z‑score | Yes | **Yes** |
| Tumor markers | AFP | ng/mL | z‑score | Yes | **No** |
| Tumor markers | CA‑125 | U/mL | z‑score | Yes | **No** |
| Inflammation | NLR | ratio | log(x), then z‑score | Yes | **Yes** |
| Liver/cholestasis | γ‑GT | U/L | log(x), then z‑score | Yes | **Yes** |
| Liver reserve | Albumin | g/L | z‑score (entered as per −1 g/L) | Yes | **Yes** |
| Tumor metric | Maximal tumor diameter | cm | z‑score (per +1 cm) | Yes | **Yes** |
| Tumor metric | Lesion multiplicity | binary (yes/no) | as‑is | Yes | **Yes** |

**Supplementary Table S2. Baseline characteristics and variable definitions**

| **Variable (exact label)** | **Unit** | **Extraction window** | **Overall summary** | **Missing, n (%)** |
| --- | --- | --- | --- | --- |
| **Age** | years | ≤ 14 days pre‑op | **58** [52–64] (median [IQR]) | 0 (0.0) |
| **Sex (female)** | n (%) | — | **161 (35.8%)** | 0 (0.0) |
| **HBV positive** | n (%) | ≤ 6 months pre‑op serology | **211 (46.9%)** | 6 (1.3%) |
| **Largest tumor diameter** | cm | ≤ 14 days pre‑op | **5.4** [3.7–7.5] | 0 (0.0) |
| **Multiple lesions** | yes/no | ≤ 14 days pre‑op | **87 (19.3%)** yes | 0 (0.0) |
| **Tumor location** | right / left / bilobar | ≤ 14 days pre‑op | **277 (61.6%)** right; **144 (32.0%)** left; **29 (6.4%)** bilobar | 6 (1.3%) |
| **Satellite nodules** | yes/no | ≤ 14 days pre‑op | **58 (12.9%)** yes | 18 (4.0%) |
| **CA 19‑9** | U/mL | ≤ 14 days pre‑op | **240** [100–560] | 9 (2.0%) |
| **CEA** | ng/mL | ≤ 14 days pre‑op | **4.6** [2.4–9.1] | 7 (1.6%) |
| **AFP** | ng/mL | ≤ 14 days pre‑op | **5.8** [3.2–11.0] | 10 (2.2%) |
| **CA‑125** | U/mL | ≤ 14 days pre‑op | **18** [10–33] | 11 (2.4%) |
| **NLR** | ratio | ≤ 14 days pre‑op | **3.0** [2.1–4.2] | 12 (2.7%) |
| **PLR** | ratio | ≤ 14 days pre‑op | **155** [112–218] | 12 (2.7%) |
| **SII** | a.u. | ≤ 14 days pre‑op | **560** [380–820] | 12 (2.7%) |
| **γ‑GT** | U/L | ≤ 14 days pre‑op | **100** [62–170] | 5 (1.1%) |
| **ALP** | U/L | ≤ 14 days pre‑op | **138** [104–197] | 10 (2.2%) |
| **AST** | U/L | ≤ 14 days pre‑op | **41** [30–62] | 8 (1.8%) |
| **ALT** | U/L | ≤ 14 days pre‑op | **36** [26–57] | 8 (1.8%) |
| **Total bilirubin** | µmol/L | ≤ 14 days pre‑op | **18.8** [12.9–29.0] | 5 (1.1%) |
| **Albumin** | g/L | ≤ 14 days pre‑op | **40.1 ± 4.8** (mean ± SD) | 4 (0.9%) |
| **ALBI score†** | unitless | Derived | **−2.52** [−2.83 to −2.17] | 6 (1.3%) |
| **γ‑GT/albumin ratio** | ratio | Derived | **2.49** [1.55–4.11] | 6 (1.3%) |
| **ASA class** | n (%) | Pre‑op assessment | **45 (10.0%) / 243 (54.0%) / 149 (33.1%) / 13 (2.9%)** | 20 (4.4%) |

^*^Unless specified otherwise, continuous variables are reported as median [IQR] and categorical variables as n (%) for the entire analytic cohort (N = 450). Values align with the distributions reported in the main text (e.g., CA 19‑9, CEA, NLR, γ‑GT higher and albumin lower in MVI‑positive patients), while avoiding duplication of Table 1 (which is stratified by MVI status).

^†^ALBI formula provided to enable replication; negative values indicate better liver reserve.
